# Hygrothermal Treatment Improves the Dimensional Stability and Visual Appearance of Round Bamboo

**DOI:** 10.3390/polym17060747

**Published:** 2025-03-12

**Authors:** Tong Tang, Changhua Fang, Zhen Sui, Chuanle Fu, Xuelin Li

**Affiliations:** 1 School of Art & Design, Qilu University of Technology (Shandong Academy of Sciences), Jinan 250353, China; tangtong@qlu.edu.cn (T.T.);; 2 Key Laboratory of Bamboo and Rattan Science and Technology of the State Forestry Administration, Department of Biomaterials, International Center for Bamboo and Rattan, Beijing 100102, China

**Keywords:** round bamboo, hygrothermal treatment, dimensional stability, range analysis, color

## Abstract

Green, newly felled bamboo stems are prone to cracking during the drying process due to the growth stress and moisture gradient. To improve the drying quality and dimensional stability of bamboo stems, this study applied hygrothermal treatment under atmospheric pressure to newly felled bamboo stems. The temperature, relative humidity, and duration of the treatment were optimized using an orthogonal L_9_ (3^4^) experimental design. The results show that the surface color of round bamboo became more uniform after hygrothermal treatment. Furthermore, hygrothermal treatment could reduce the cuticular wax and silicon layer detachment on the surface of round bamboo after drying. According to the range and variance analysis, the relative humidity had the greatest impact on dimensional stability, followed by treatment duration, whereas the temperature had a limited effect. The swelling rate of round bamboo under a hygrothermal treatment at a relative humidity of 95%, a temperature of 95 °C, and a duration of 3 h was decreased 53.72% and 62.76% compared with untreated round bamboo after moisture or water absorption for 7 d, respectively. However, no significant difference was observed in the color of the round bamboo under different hygrothermal treatment conditions. Overall, this study suggests that hygrothermal treatment could be a highly promising technology for improving the dimensional stability of newly felled bamboo stems.

## 1. Introduction

Bamboo is a degradable biomass material that can sustainably grow once planted. It only takes 3–5 years to grow into a useful material, and more than 3.2 × 10^7^ hm^2^ of bamboo forests have been reported worldwide [1]. Bamboo has been used in the field of buildings, bridges, urban pipes, and packing for its excellent mechanical properties. However, bamboo is prone to deformation during use, resulting in reduced safety, durability, and esthetics. The modification methods used to improve the dimensional stability of bamboo and to prevent it cracking can be summarized as drying treatments, thermal treatments, modification of the cell wall structure, surface coating, and densification [2,3,4,5,6].

The round bamboo culm is covered by a cortex and pith ring. The waxy and silica layer on the bamboo cortex surface forms a natural barrier, and the outer-layer cells of the cortex have few pits [7]. The bamboo pith ring lacks vascular tissue but consists of specialized cells. As these cells mature, their walls progressively thicken and lignify, eventually differentiating into stone cells. Stone cells exhibit a dense, hardened structure with multilayered cell walls [8,9]. Additionally, the main transverse transport channels of round bamboo are the pits on the side walls in the internode and the deflecting vascular bundles in the node [10,11]. Therefore, the permeability of the modifier in round bamboo is lower than that in the boards, and the adhesion of the coating on the surface of round bamboo is also worse than that on the boards. At present, drying and thermal treatment are among the most commonly used methods to solve the deformation and cracking problems of round bamboo.

A newly felled bamboo stem has a high moisture content (45–90%) and growth moisture gradient, which varies based on species, age, and environmental conditions [12]. The moisture content of the middle layer is higher than the pith ring and cortex in freshly cut bamboo and this variation significantly influences the drying behavior and mechanical properties of bamboo during processing [8]. To avoid damage by microbes, newly felled bamboo requires immediate drying before further storage, transport, processing, modification, and utilization. In the process of drying treatment, the stress and moisture gradients of bamboo gradually decreased with the evaporation of moisture, leading to the improvement of the bamboo’s dimensional stability. However, bamboo drying usually takes a long time. For example, the natural drying and kiln drying of round bamboo take about 6–12 weeks and 3–6 weeks, respectively [13].

The hygrothermal treatment of wood is one of the most prominent thermal treatment techniques, and is a cost-effective and environmentally friendly technique that can be used to improve the dimensional stability, durability, and biological resistance of wood [14]. Hygrothermal treatment prior to drying has been applied to treat tree disks and square timbers to decrease cracking and improve drying speed [15,16,17], and may be a promising method for the treatment of newly felled bamboo stems. Bamboo and wood are primarily composed of cellulose, hemicellulose, and lignin. However, they differ in anatomical structure, chemical composition, mechanical properties, and dimensional stability. As a result, research findings on wood may not directly apply to bamboo. Previous studies have primarily focused on applying hygrothermal treatment to dried round bamboo, while research on treating freshly felled round bamboo remains limited. Hygrothermally treating round bamboo with saturated steam at a temperature of 140–180 °C before drying it in a kiln can improve its quality [18]. However, the high temperatures of hygrothermal treatment induce the degradation of bamboo materials, resulting in an increase in mass loss and a decrement of bending strength [19,20].

To enhance the dimensional stability and drying quality of bamboo, freshly felled bamboo culms were subjected to hygrothermal treatment at temperatures below 100 °C. This study examined the effects of the treatment on dimensional stability and surface color. Hygrothermal treatment under atmospheric pressure presents a simple and cost-effective approach for improving bamboo’s properties. The dimensional stability of bamboo was characterized by moisture swelling, water swelling, and shrinkage. Based on the results of range and variance analysis, the parameters of the hygrothermal treatment were optimized. Moreover, the surface color of round bamboo was measured to explore the hygrothermal treatment in terms of visual appearance. These results contribute to the promotion and application of hygrothermal treatment for fresh round bamboo, which would also provide a new path toward optimizing the drying technology of round bamboo.

## 2. Materials and Methods

### 2.1. Materials

Moso bamboo [*Phyllostachys edulis* (Carr.) J. Houz], 5 years old, was obtained from Huangshan, Anhui Province, China (118°1′ E, 30°1′ N). Moso bamboo specimens from 1.5 m (height from the base) to 3.5 m were used in this study, with a diameter at breast height of about 10 cm.

### 2.2. Sample Preparation

**Figure 1:**
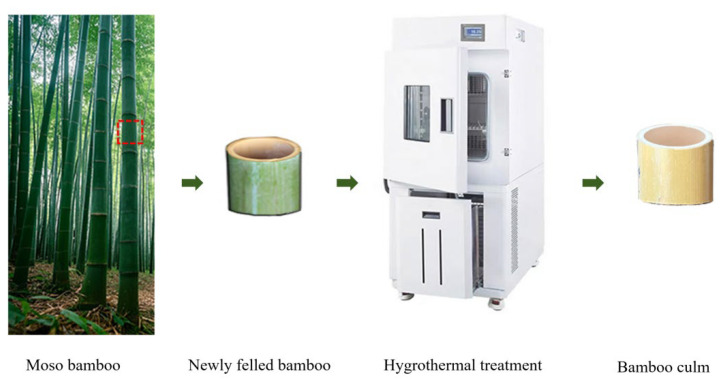
Schematics of sample preparation.

The newly felled bamboo had an initial moisture content of approximately 75%. After removing the nodes, the bamboo was processed into culms with a height of 3 cm. Subsequently, the round bamboo was hygrothermally treated in a high- and low-temperature humidity test chamber (BPHS-060A, Shanghai Yiheng Scientific Instrument Co., Ltd., Shanghai, China) under different conditions (Figure 1). Finally, the round bamboo was conditioned in the chamber at 65 ± 5% of relative humidity and a temperature of 20 ± 2 °C until reaching an equilibrium moisture content.

To investigate the synergistic effects of temperature, relative humidity, and the duration of hygrothermal treatment on the dimensional stability and visual appearance of round bamboo, an orthogonal experiment L_9_ (3^4^) was designed (Table 1). Some studies have shown that the aggregation of water molecules within molecular chains lowers the glass transition temperature of lignin from 160 °C to 60–95 °C [21,22,23,24,25]. Therefore, the constituents of bamboo may degrade under a high relative humidity at temperatures below 100 °C. Considering the initial moisture content of freshly felled bamboo and upon exploring the effects of hygrothermal treatment on chemical structure, the treatment parameters of temperature (75 °C, 85 °C, and 95 °C), relative humidity (45%, 70%, and 95%), and duration (1 h, 3 h, and 6 h) were selected for this study.

### 2.3. Characterizing Fourier Transform Infrared (FTIR) Spectra

The chemical structure of bamboo was obtained by FTIR (Nicolet iN10, Thermo Scientific, Waltham, MA, USA) spectra over the wavenumber range of 500–4000 cm^−1^. The FTIR spectra were obtained using dried bamboo powder, which was pressed into a pellet with KBr, at a resolution of 4 cm^−1^.

### 2.4. Dimensional Stability

The round bamboo after hygrothermal treatment was conditioned in a high- and low-temperature humidity test chamber at a relative humidity of 65 ± 5% and a temperature of 20 ± 2 °C to reach an moisture content equilibrium. Subsequently, the samples were placed in a high- and low-temperature humidity test chamber at a relative humidity of 100 ± 5% and a temperature of 20 ± 2 °C, and the thickness of moisture swelling in the walls of the samples was calculated at 2 d, 7 d, and 9 d, respectively. Furthermore, the thickness of the water swelling in the walls of the round bamboo samples was also calculated at 7 d and 60 d from the moisture content equilibrium to water saturation, respectively.

Additionally, the shrinkage rate of the wall thickness of the round bamboo samples was calculated from water saturation to the moisture content equilibrium (with conditioning at a relative humidity of 65 ± 5% of and a temperature of 20 ± 2 °C). The dimension changes in the round bamboo during water loss also need to be considered in the study of dimensional stability. For example, in the process of water loss, bamboo boards may become deformed, and the compressed wood may undergo deformation recovery [20]. The change rate of the wall thickness of water-saturated round bamboo after water loss for 7 d compared to its initial state (moisture content equilibrium) was calculated. All the measurements were repeated 40 times.

### 2.5. Visual Appearance Quantification of Round Bamboo

The visual appearance of the round bamboo samples was evaluated by surface color using a chroma meter (CC-6834, BYK-Gardner, Grazrid, Germany), referring to the color space CIE L*a*b*. CIE L*a*b* is defined by the International Commission on Illumination, the L* (lightness), a* (red–green coordinates), b* (yellow–blue coordinates), and C* (color saturation) were measured using a D65 illuminant and a 10° standard observer. The color changes in the round bamboo samples were calculated in terms of the difference before and after hygrothermal treatment. For example, Δ*a* = a*_after_ − a*_before_. To reduce experimental errors, the measurement was performed 20 times. The images of the round bamboo samples were captured on a mobile phone (iPhone 13, Apple, Cupertino, CA, USA).

### 2.6. Statistical Analysis

The statistical analysis was performed using the IBM SPSS Statistics 23 software, which is based on the least significant difference (LSD) with a significance level (*p*) of 0.05. The range analysis was used to assess the impact weights of different treatment factors, which determines the optimal combination of hygrothermal conditions. The K-value represents the sum of indicators at each level, while the R-value is the maximum K-value minus the minimum K-value under the same factor, reflecting the magnitude of the factor’s impact on the results. Furthermore, Duncan’s multiple comparison analysis was used to analyze the significance level of different treatment parameters affecting the dimensional stability of round bamboo and to verify the scientific validity of the optimal combination of hygrothermal conditions.

## 3. Results and Discussion

### 3.1. Chemical Structure of Round Bamboo

**Figure 2:**
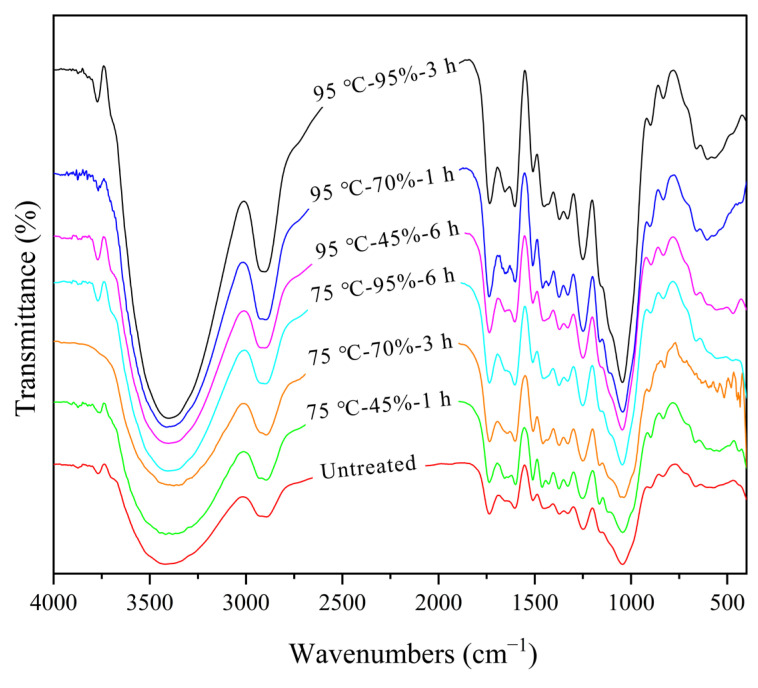
FTIR spectra of bamboo after hygrothermal treatment.

The chemical structure of the cell wall influences the dimensional stability of bamboo [26]. Therefore, the chemical structure of round bamboo after hygrothermal treatment was characterized, and the FTIR spectra showed that the intensity of some peaks was changed (Figure 2). The peak at 3610–3425 cm^−1^ was ascribed to hydroxyl (-OH) stretching vibrations. Water molecules establish new hydrogen bonds with the available hydroxyl group sites in lignin, hemicellulose, and the amorphous region of cellulose during the hygrothermal treatment of round bamboo [27,28]. Hydrogen bonds may also be established between the water molecules, leading to the aggregation of water molecules and the formation of multilayered water [10]. Therefore, the distance between the cell wall molecules was gradually increased and the extensibility of molecular chains was enhanced [26], thus providing the movement space of cell wall molecules. The intensity of the peak at 3610–3425 cm^−1^ was increased after hygrothermal treatment compared with untreated bamboo, indicating an increase in -OH groups during hygrothermal treatment [29]. The peak at 1745–1730 cm^−1^ was assigned to C=O stretching vibration of the acetyl groups and uronic acid ester groups in hemicellulose and the carbonyl groups in lignin. The intensity of the peak at 1745–1730 cm^−1^ of bamboo after hygrothermal treatment at 95 °C was higher than that of untreated bamboo. The peak at 895 cm^−1^ became sharper after hygrothermal treatment, and was assigned to the C-H deformation of cellulose, indicating that the molecular chains in the cellulose have been rearranged [30].

The peaks at 1510 cm^−1^ and 1600 cm^−1^ were ascribed to the C=C stretching of the aromatic skeletal vibrations in lignin [31,32]. The glass transition temperature of lignin decreases with the aggregation of water molecules in molecular chains [21,22,23,33], which decreases from 160 °C to 60–95 °C as dry wood turns into moist wood [24]. Straw or bamboo lignin is classified as a G-S-H lignin, and the glass transition temperature of wheat straw lignin is about 53–63 °C with a water content of 8.4% [25]. Therefore, the molecules of lignin in newly felled bamboo stems obtained sufficient energy to undergo glass transition under hygrothermal treatment at a temperature of 75–95 °C and a relative humidity of 70–95%. In the highly elastic state, lignin molecular chains may rearrange, increasing the intensity of C=C stretching in the aromatic skeletal vibrations. Overall, the chemical structure of the bamboo hardly degraded, but its molecular chain had been rearranged.

### 3.2. Swelling of Round Bamboo

**Figure 3:**
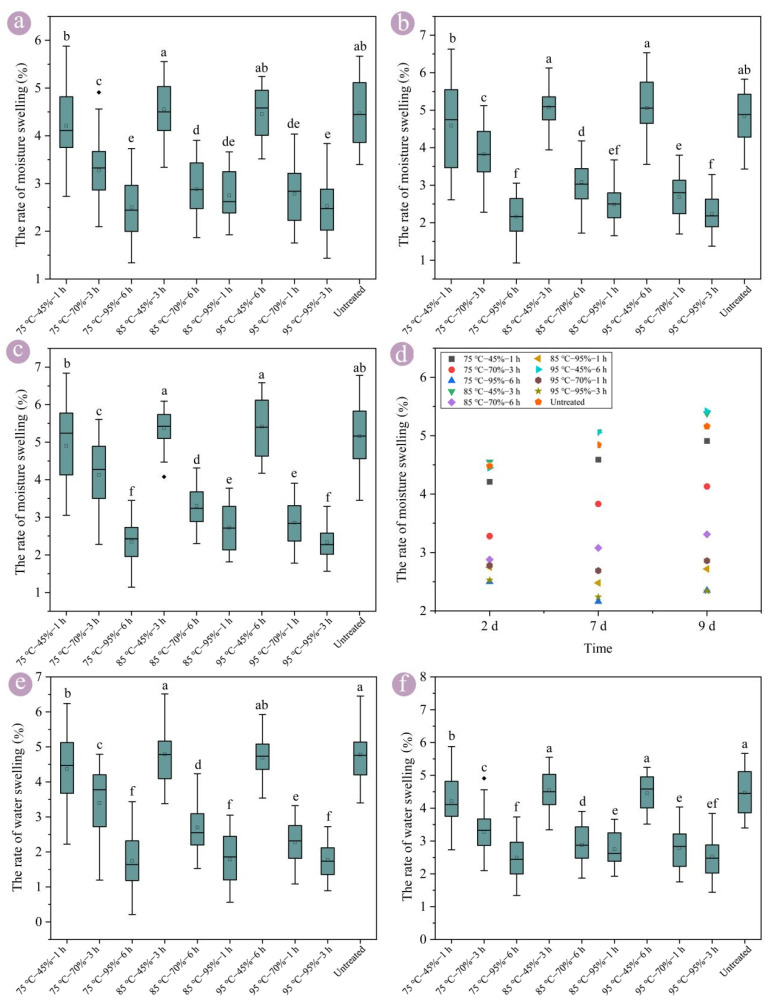
The wall swelling rate of round bamboo in a radial direction after hygrothermal treatment. (**a**) The rate of moisture swelling for 2 d, (**b**) 7 d, and (**c**) 9 d. (**d**) The scatter plot of moisture swelling. (**e**) The rate of water swelling for 7 d, (**f**) 60 d. Note: The swelling rates followed by the same superscript letters in the same figure are not significantly different.

The results show that hygrothermal treatment improves the swelling dimensional stability of round bamboo (Figure 3). The swelling rate in wall thickness of untreated round bamboo was 4.48% after moisture absorption for 2 d, whereas the swelling rates of 95 °C–95%–3 h and 75 °C–95%–6 h were only about 2.50% (Figure 3a). The difference in moisture swelling rate was gradually increased as extending the moisture absorption duration. The swelling rate of 95 °C–95%–3 h was decreased by 43.52%, 53.72%, and 54.65% compared with untreated round bamboo after moisture absorption for 2 d, 7 d, 9 d, respectively. Similarly, it decreased by 62.76% and 59.67% compared with the untreated round bamboo after water absorption for 7 d and 60 d, respectively. The swelling dimensional stability of round bamboo significantly improved after hygrothermal treatment under humidity conditions of 95%. In addition, no significant difference in the swelling dimensional stability between 95 °C–95%–3 h and 75 °C–95%–6 h conditions was observed.

Bamboo has a protective layer of waxes on its cortex, and the waxes have notable hydrophobicity and antibacterial activity [34]. In general, a large area of cuticular waxes may peel off the round bamboo after drying treatment, leading it to lose its protective effect. However, partial extractives together with water molecules gradually moved to the cortex of bamboo during hygrothermal treatment, which would join with melted waxes to form a new protective layer and improve the dimensional stability of round bamboo [35]. Additionally, under a high moisture content, the lignin and hemicellulose might undergo glass transition under temperatures of 75–95 °C [23,25,36] (Figure 3), resulting in the softening of the round bamboo. The soft round bamboo could release partial stress and balance the gradient of moisture content, which improve the quality of the dried round bamboo. Furthermore, water molecules are able to increase the fluidity and extensibility of molecular chains of microfibrils, accelerating the sliding between the microfibrils and the matrix [37], and the molecular chains can be rearranged [23], giving the round bamboo a more balanced structure and improving the dimensional stability of round bamboo.

The hygroscopicity properties of bamboo are also linked to the content of accessible -OH groups [38]. It is worth noting that the intensity of the peak at 3610–3425 cm^−1^ was increased after hygrothermal treatment compared with untreated bamboo (Figure 2), only indicating the increment of -OH groups. The changes in the -OH group content of bamboo after hygrothermal treatment were mainly due to inter-molecular hydrogen bonding and crosslinking reactions, which would decrease permeability and water absorption.

Bamboo has the characteristic of hygroscopic hysteresis [39,40]. The moisture content equilibrium of round bamboo treated at 45% was lower than that treated under 95% humidity conditions, indicating that it might have a higher swelling rate during moisture or water absorption processes (Figure 3). The round bamboo hygrothermally treated at a relative humidity of 45% was more like that subjected to the drying process. Therefore, the round bamboo hygrothermally treated under a humidity of 45% was not significantly different to the untreated bamboo (Figure 3).

### 3.3. Shrinkage of Round Bamboo

**Figure 4:**
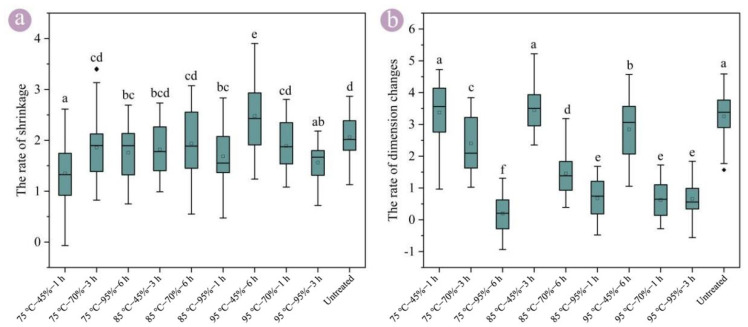
The wall shrinkage rate of round bamboo in a radial direction after hygrothermal treatment. (**a**) Water loss for 7 d compared to the water-saturated state; (**b**) water loss for 7 d compared to the initial equilibrium state. Note: The shrinkage rates followed by the same superscript letters in the same figure are not significantly different at <0.05.

The dimensional stability of the shrinkage of round bamboo was slightly improved after hygrothermal treatment (Figure 4a), which might be due to the difficulty of water evaporation caused by the increased new protective layer. The hygrothermal treatment conditions had a limited impact on the rate of shrinkage compared with swelling. The rate of shrinkage exhibited no significant difference between 75 °C–45%–1 h and 95 °C–95%–3 h, although the rate of swelling under the 75 °C–45%–1 h conditions was significantly higher than that under 95 °C–95%–3 h. The hygrothermally treated round bamboo at 95% was basically recovered to an equilibrium state after water loss for 7 d, and exhibited a better dimensional stability (Figure 4b).

The water in bamboo could be classified as either free water or bound water, and the bound water has profound effects on the dimensional stability of bamboo [41]. In the initial stage of bamboo’s water loss, the surface water evaporates and the moisture gradient forms. The capillary effects could play a major role as the water in bamboo moves from high to low water content areas [42]. Subsequently, the transfer between bound water and free water could be a significant power source in water loss at a temperature of 20 °C [43]. As the cell wall shrinks, the pore volume generated by water loss gradually decreases, which restores the morphology of the cell. The rate of shrinkage under 95 °C–45%–6 h conditions was even higher than that of the untreated bamboo, which might be due to the extractives being volatilized with water molecules, leading to the enhanced permeability of the pit membrane and the decreased resistance of water transportation among the cells [44]. Additionally, the lignin molecular chains under 95 °C–45%–6 h were not in a glass transition, and were not rearranged.

### 3.4. Optimization of Modification Conditions

In the orthogonal experiments, the swelling dimensional stability of bamboo after hygrothermal treatment at relative humidities of 70% and 95% was significantly improved, whereas that at a relative humidity of 45% had no significant difference compared to the untreated round bamboo (Figure 3). The results show the swelling rates of the experiments 75 °C–95%–6 h and 95 °C–95%–3 h was significantly lower than that of 95 °C–45%–6 h, although the 95 °C–45%–6 h and 75 °C–95%–6 h experiments have the same modification duration, and the 95 °C–45%–6 h and 95 °C–95%–3 h experiments have the same modification temperature. Therefore, the primary and secondary order of different treatment factors on the dimensional stability of round bamboo were analyzed through range and variance analysis, and the optimal modification conditions were screened.

The primary and secondary order of hygrothermal treatment factors on the swelling dimensional stability was calculated by the R-value in accordance with the range analysis method, which was as follows: humidity > duration > temperature (Table 2). With an increase in the relative humidity of the hygrothermal treatment, the swelling dimensional stability of round bamboo was improved. The swelling dimensional stability of the hygrothermally treated round bamboo at a temperature of 95 °C was significantly better than that at temperatures of 75 °C and 85 °C, but the difference between the temperatures of 75 °C and 85 °C was not statistically significant.

Notably, based on the K-value calculated using the range analysis method, it was found that extending the treatment duration actually reduced the swelling dimensional stability of round bamboo. In the process of hygrothermal treatment, partial extractives together with water molecules gradually moved to the cortex of the bamboo and consecutive pore paths formed as the extractives moved [44,45], resulting in the increased moisture or water permeability of round bamboo and its reduced dimensional stability. Therefore, the hygrothermal treatment of round bamboo for 1 h would result in a better dimensional stability than treatment for 3 h and 6 h. The optimal hygrothermal conditions that improve the swelling dimensional stability of round bamboo were a temperature of 95 °C, a relative humidity of 95%, and a duration of 1 h. For the shrinkage dimensional stability of round bamboo, the optimal hygrothermal conditions were still a relative humidity of 95% and a duration of 1 h.

To further verify the scientific validity of the selection of hygrothermal treatment conditions, the significance of the effects of hygrothermal treatment factors and their interactions can be clarified by variance analysis based on the results of the range analysis. The variance analysis of the moisture swelling rate revealed that no significant difference in modification temperature parameters (75 °C, 85 °C, and 95 °C) on the dimensional stability of the round bamboo after moisture absorption for 2 d and 7 d was observed (Table 3). When the round bamboo absorbed moisture for 9 d, the dimension of the round bamboo reached a relatively stable state. Moreover, the round bamboo exhibited a better dimensional stability with hygrothermal treatment at 95 °C. Therefore, the preferred modification temperature was still 95 °C. The humidity of the treatment was selected as 95%, based on the moisture absorption, swelling rate, and the range analysis results. The modified treatment time was selected as 1 h from the perspective of energy conservation and emission reduction, as well as the moisture absorption, swelling rate, and range analysis results.

### 3.5. Visual Appearance of Round Bamboo

**Figure 5:**
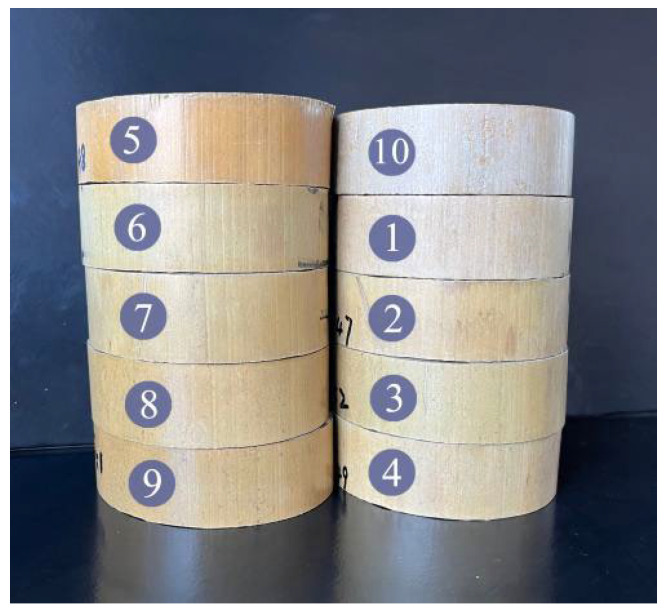
An image of round bamboo (the numbers marked on the image represent the number of the experiment).

The cuticular wax and silicon on bamboo culm form a natural protective layer during growth, which has notable hydrophobicity and antibacterial properties [34,46,47]. However, the cuticular wax and silicon on bamboo culm exhibit a dull gray color and might be bulged or detached after natural drying, resulting in a lower-quality appearance (Figure 5). To maintain the excellent visual appearance of round bamboo, microwave vacuum drying or green preservation techniques have been used to treat bamboo [48,49]. That hygrothermal treatment improves the dimensional stability of round bamboo has been verified in our study. If hygrothermal treatment can also improve the visual appearance of round bamboo, this will be more conducive to the promotion of hygrothermal treatment technology.

The visual appearance of round bamboo samples was evaluated after hygrothermal treatment. The results showed that the fresh round bamboo was hardly cracked and had a smooth surface after hygrothermal treatment and the subsequent drying process, which results in a higher-quality appearance compared with the naturally dried bamboo (Figure 5). The L* of round bamboo was decreased after hygrothermal treatment, whereas the a*, b*, and C* were increased compared with the untreated round bamboo (Table 4). The color of bamboo is mainly related to the chemical structures of the lignin and extractives [50]. In the process of hygrothermal treatment, the extractives, including phenolic, alcohol, and aldehyde compounds, migrate to the bamboo surface with water molecules [51], and the compounds are oxidized during hygrothermal treatment [52,53], leading to an increased a* and b* of round bamboo and a decreased L*. Additionally, the curled molecular chains in the lignin undergo relaxation and begin to disintegrate to form mobile fragments when the glass transition slowly takes place [54]. The connecting bonds between some of lignin’s basic units are broken and undergo oxidation reactions, which generates a mixed system of unsaturated ketones and quinones, and increases the value of b* [23,55,56]. With the further oxidation of quinone structures, orthoquinone structures are formed, resulting in a decrease in the L* value.

The color changes in the 75 °C–95%–6 h and 95 °C–95%–3 h experiments were greater than in 75 °C–45%–1 h, which was mainly due to the glass transition of lignin at a relative humidity of 95% and the accumulation of extractives on the surface of the bamboo with the extension of the duration. No significant difference in surface color among experiments Nos. 2–9 with L* values ranging from 54.39 to 59.28, a* values ranging from 7.28 to 9.77, b* values ranging from 25.08 to 26.68, and C* values ranging from 26.30 to 28.28 was found. Therefore, considering the comprehensive impact of hygrothermal treatment on the dimensional stability and visual appearance of round bamboo, 95 °C–95%–1 h are the optimal hygrothermal condition.

## 4. Conclusions

Hygrothermal treatment is an effective method to improve the dimensional stability and visual appearance of round bamboo, which is a modification technology worthy of industrial promotion. Fresh round bamboo was hardly cracked and the surface color was more uniform after hygrothermal treatment and the subsequent drying process, which has a better appearance quality compared with the naturally dried bamboo. In the process of hygrothermal treatment, partial extractives would join with melted waxes to form a new protective layer. Furthermore, the problem of cuticular wax and silicon layer detachment on the surface of round bamboo after drying was solved through hygrothermal treatment. Relative humidity had the greatest impact on dimensional stability, followed by treatment duration, and the effect of temperature was relatively limited. In a state of a high moisture content, the lignin and hemicellulose might enter into the glass state under temperatures of 75–95 °C, releasing partial growth stress and rearranging molecular chains. Round bamboo was subjected to hygrothermal treatment under a relative humidity of 95%, and its swelling rate decreased by over 50% after moisture or water absorption for 7 d, and even essentially returned to its initial equilibrium value after 7 d of dehydration from its water-saturated state. Considering the dimensional stability and visual appearance of round bamboo, the optimal hygrothermal treatment conditions are a temperature of 95 °C, a relative humidity of 95%, and a duration of 1 h.

## Figures and Tables

**Table 1:** Orthogonal experimental design named L_9_ (3^4^).

Experiment No.	Definition	Temperature (°C)	Relative Humidity (%)	Duration (h)
1	75 °C–45%–1 h	75	45	1
2	75 °C–70%–3 h	75	70	3
3	75 °C–95%–6 h	75	95	6
4	85 °C–45%–3 h	85	45	3
5	85 °C–70%–6 h	85	70	6
6	85 °C–95%–1 h	85	95	1
7	95 °C–45%–6 h	95	45	6
8	95 °C–70%–1 h	95	70	1
9	95 °C–95%–3 h	95	95	3
10	Untreated	Untreated round bamboo served as the control group		

**Table 2:** The range analysis of different factors affecting the dimension stability of round bamboo.

Dimensional Stability	Index	Factors	K_1_	K_2_	K_3_	R-Value	Primary and Secondary Order	Optimal Combination
The rate of moisture swelling	2 d	Temperature (A)	3.33	3.39	3.26	0.13	B > C > A	A_3_B_3_C_1_
		Relative humidity (B)	4.41	2.98	2.59	1.82		
		Time (C)	3.25	3.45	3.28	0.20		
	7 d	Temperature (A)	3.53	3.55	3.33	0.22	B > C > A	A_3_B_3_C_1_
		Relative humidity (B)	4.91	3.20	2.29	2.62		
		Time (C)	3.25	3.71	3.43	0.46		
	9 d	Temperature (A)	3.80	3.80	3.54	0.26	B > C > A	A_3_B_3_C_1_
		Relative humidity (B)	5.23	3.43	2.47	2.76		
		Time (C)	3.49	3.95	3.69	0.46		
The rate of water swelling	7 d	Temperature (A)	3.17	3.09	2.91	0.26	B > C > A	A_3_B_3_C_1_
		Relative humidity (B)	4.62	2.79	1.77	2.85		
		Time (C)	2.81	3.32	3.04	0.51		
	60 d	Temperature (A)	3.69	3.71	3.40	0.31	B > C > A	A_3_B_3_C_1_
		Relative humidity (B)	5.21	3.41	2.19	3.02		
		Time (C)	3.27	3.92	3.61	0.65		
The rate of shrinkage	7 d	Temperature (A)	1.666	1.811	1.988	0.32	C > A > B	A_1_B_3_C_1_
		Relative humidity (B)	1.888	1.899	1.677	0.22		
		Time (C)	1.644	1.755	2.066	0.42		

Note: A1 = 75 °C, A2 = 85 °C, A3 = 95 °C, B1 = 45%, B2 = 70%, B3 = 95%, C1 = 1 h, C2 = 3 h, C3 = 6 h.

**Table 3:** The variance analysis of the dimensional stability of round bamboo.

Dimensional Stability	Index	Factors	f	Sum of Square	Mean Square	F	P	Significance
The rate of moisture swelling	2 d	Temperature (A)	2	1.035	0.518	1.308	0.272	
		Relative humidity (B)	2	197.006	98.503	249.051	0.000	*
		Time (C)	2	2.610	1.305	3.299	0.038	*
	7 d	Temperature (A)	2	3.106	1.553	2.958	0.053	
		Relative humidity (B)	2	380.292	190.146	362.168	0.000	*
		Time (C)	2	11.730	5.865	11.171	0.000	*
	9 d	Temperature (A)	2	4.969	2.485	4.589	0.011	*
		Relative humidity (B)	2	425.166	212.583	392.581	0.000	*
		Time (C)	2	11.189	5.595	10.332	0.000	*
The rate of water swelling	7 d	Temperature (A)	2	3.769	1.884	3.109	0.046	*
		Relative humidity (B)	2	449.101	224.551	370.515	0.000	*
		Time (C)	2	14.246	7.123	11.753	0.000	*
	60 d	Temperature (A)	2	6.608	3.304	4.518	0.012	*
		Relative humidity (B)	2	498.144	249.072	340.567	0.000	*
		Time (C)	2	22.590	11.295	15.444	0.000	*
The rate of shrinkage	7 d	Temperature (A)	2	5.565	2.783	8.113	0.000	*
		Relative humidity (B)	2	3.504	1.752	5.109	0.007	*
		Time (C)	2	10.137	5.069	14.778	0.000	*

Note: An asterisk (*) indicates that different parameter values of the influencing factor in the same row have a significant impact on dimensional stability (*p* < 0.05).

**Table 4:** The color of round bamboo after hygrothermal treatment.

Experiment No.	L*	a*	b*	C*
1 (75 °C–45%–1 h)	58.14 (2.85)	4.80 (1.57)	22.92 (2.89)	23.46 (2.94)
2 (75 °C–70%–3 h)	56.95 (2.18)	7.88 (1.25)	25.08 (1.94)	26.30 (2.06)
3 (75 °C–95%–6 h)	56.06 (1.53)	7.60 (0.73)	26.60 (2.70)	27.67 (2.74)
4 (85 °C–45%–3 h)	59.28 (1.64)	7.28 (1.28)	25.69 (2.11)	26.73 (2.19)
5 (85 °C–70%–6 h)	56.58 (1.56)	9.63 (1.69)	26.55 (1.69)	28.28 (1.90)
6 (85 °C–95%–1 h)	56.30 (1.21)	7.65 (0.92)	25.98 (1.86)	27.75 (2.30)
7 (95 °C–45%–6 h)	57.13 (1.38)	7.73 (1.14)	26.42 (1.97)	27.69 (2.13)
8 (95 °C–70%–1 h)	57.80 (1.94)	7.69 (1.13)	26.68 (2.79)	27.79 (2.78)
9 (95 °C–95%–3 h)	54.39 (1.29)	9.77 (1.18)	26.19 (2.46)	27.96 (2.64)
10 (Untreated)	63.22 (2.37)	2.00 (0.81)	18.36 (2.56)	18.48 (2.55)

Note: Values in parentheses are standard deviations.

## Data Availability

Data are contained within the article.
